# The Immune Regulatory Functions of CD226 and Its Implications in Immune-Mediated Diseases

**DOI:** 10.3390/biom15071007

**Published:** 2025-07-14

**Authors:** Keyan Liu, Yuanzhen Liu, Huabao Xiong, Zhaochen Ning

**Affiliations:** 1 Department of Public Health, Jining Medical University, Jining 272067, China; liukeyan@mail.jnmc.edu.cn; 2 Jining Key Laboratory of Immunology, Jining Medical University, Jining 272067, China; liuyuanzhen@stu.mail.jnmc.edu.cn

**Keywords:** CD226, tumor immunity, infectious diseases, autoimmune diseases, immune regulation

## Abstract

CD226, a member of the immunoglobulin superfamily, serves as a critical regulator in various immunological processes. CD226 is expressed across immune and non-immune cells, with predominant expression being observed in natural killer (NK) cells and T cells. By engaging ligands CD155 and CD112, it orchestrates diverse signaling pathways that modulate T cell differentiation and effector functions while enhancing NK cell activation and cytotoxicity. Genetic polymorphisms and the dysregulated expression of CD226 are closely associated with susceptibility to autoimmune diseases, infectious diseases, allergic diseases, and cancer progression. Growing evidence highlight CD226’s emerging promise as a therapeutic target for immune-mediated diseases. The present work aims to review the current understanding of CD226’s role in immune responses and to comprehensively outline its multifaceted involvement in different immunological diseases, providing insights for future research to advance our mechanistic understanding of its roles in disease pathogenesis.

## 1. Introduction

CD226 is encoded by a conserved gene located on chromosome 18 in humans [1]. The protein comprises a leader sequence, two extracellular V-set Ig-like domains (D1 and D2), a transmembrane domain, and a cytoplasmic tail [2,3,4]. The binding interactions between CD226 and its ligands are predominantly governed by a conserved “lock-and-key” motif located within the D1 domain [5]. The cytoplasmic domain contains evolutionarily conserved tyrosine (Y322) and serine (S329) residues in humans (S326 and Y319 in mice), which undergo phosphorylation upon ligand engagement to initiate downstream signaling. CD226 exhibits broad expression across immune and non-immune cells. In humans and mice, its expression is detected on NK cells, T cells, natural killer T (NKT) cells, dendritic cells, B cells, endothelial cells, monocytes/macrophages, mast cells, hematopoietic precursor cells, and megakaryocyte/platelet lineages [6,7].

**Figure 1:**
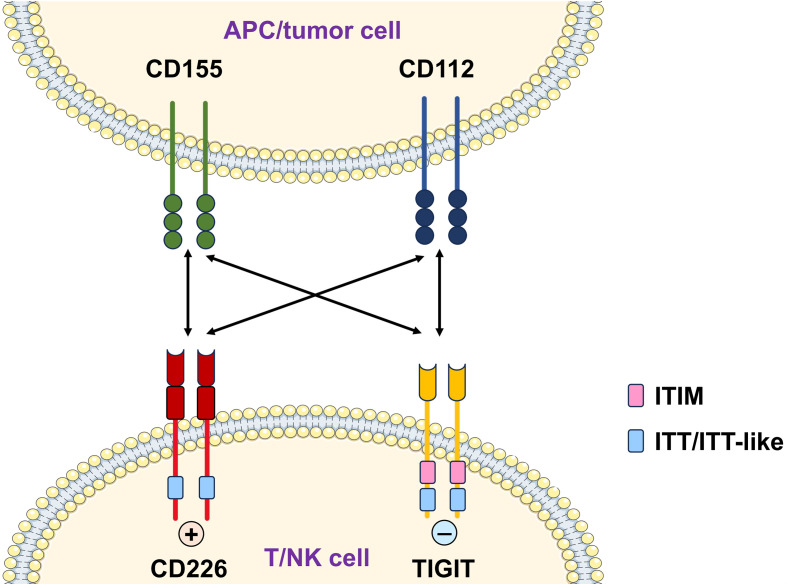
Interactions of CD226 and TIGIT with shared ligands. CD226 and TIGIT are immunoregulatory receptors, primarily expressed on T cells and NK cells, that interact with shared ligands CD112 and CD155. These ligands are often displayed on antigen-presenting cells and transformed cells. Upon ligand binding, CD226 mediates activating signals via its immunoglobulin tyrosine tail (ITT)/ITT-like motif, whereas TIGIT delivers inhibitory signals through ITIM and ITT-like motifs.

CD226 binds to CD155 (PVR) and CD112 (nectin-2), members of the nectin/nectin-like family. CD155 exhibits a higher affinity for CD226 than CD112 [8]. CD112 and CD155 exhibit expression across a wide range of cell types, including antigen-presenting cells, epithelial cells, fibroblasts, endothelial cells, neurons, pathogen-infected cells, and numerous types of cancer cells [9,10]. Notably, CD112 and CD155 also engage inhibitory receptor T cell immunoreceptor with Ig and ITIM domains (TIGIT), creating a competitive axis where CD226’s lower ligand affinity allows for dynamic immune regulation [11]. The opposing signaling of CD226 and TIGIT upon engagement with their shared ligands is illustrated in Figure 1. Upon ligand binding to CD155 or CD112, protein kinase C (PKC) phosphorylates the S329 residue of CD226, enabling its association with lymphocyte function-associated antigen-1 (LFA-1) [12]. This interaction facilitates the subsequent phosphorylation of the Y322 residue by the Src family kinase Fyn [13]. The activated CD226 initiates downstream signaling through the phosphorylation of vav guanine nucleotide exchange factor 1 (Vav1) and lymphocyte cytosolic protein 2 (LCP2), which stimulate phospholipase C gamma 2 (PLCγ2) activity. Consequently, extracellular signal-regulated kinase (ERK) and protein kinase B (PKB/AKT) are activated [2], thereby driving calcium mobilization and degranulation—critical processes for effector cell functions [1]. The strategic positioning of CD226 within this ligand–receptor network underscores its role as a pivotal immune checkpoint. The dysregulation of CD226 signaling—through genetic polymorphisms, altered expression, or imbalanced ligand interactions—disrupts immune homeostasis and is mechanistically linked to aberrant inflammation, autoimmunity, and impaired pathogen/tumor surveillance. Consequently, understanding CD226’s molecular interactions is essential for deciphering its pathophysiological roles in immune-mediated diseases.

## 2. Effects of CD226 in Immune Cells

### 2.1. CD226 and T Cells

CD226 serves as an important regulator governing the maturation and differentiation of T cells. During thymocyte development, CD226 expression increases from the double-positive to single-positive stages, with a higher expression level in CD8^+^ T cells. CD226 deficiency impairs T cell receptor signaling by reducing p38, AKT, ERK, and nuclear factor-kappa B (NF-κB) phosphorylation, leading to enhanced apoptosis [14]. Upon engagement, CD226 induces tyrosine phosphorylation of VAV1, which subsequently amplifies T cell receptor (TCR)-driven ERK activation and specifically promotes interleukin (IL)-17 production in CD4^+^ T cells [15]. The CD155-CD226 interaction further modulates memory-like CD8^+^ T cell generation in the thymus by regulating IL-4 levels and invariant NKT (iNKT) cell differentiation [16,17]. CD226 drives the differentiation of proinflammatory T helper (Th) subsets. It promotes Th17 and Th1 polarization by enhancing IL-17 and IFN-γ production, while its knockdown or blockade shifts the balance toward Th2 responses (elevated IL-4 and IL-13) [18,19]. CD226 also facilitates LFA-1-mediated costimulation in naive T cells, supporting Th1 differentiation and proliferation [20]. In T follicular helper (Tfh) cells, CD226 promotes early differentiation and proliferation, while its role diminishes in mature germinal center-Tfh cells [21]. These findings highlight CD226’s critical role in shaping the T cell repertoire and ensuring functional competence.

CD226 polymorphisms influence T cell function and disease susceptibility. The CD226-307Ser risk variant enhances interferon-γ (IFN-γ) signaling in CD8^+^ T cells via increased ERK1/2 and signal transducer and activator of transcription (STAT) 4 phosphorylation, contributing to chronic inflammation in conditions like multiple sclerosis [22]. Additionally, the TT genotype of rs763361 reduces CD226 expression on T cells while elevating IL-17A secretion, linking it to autoimmune dysregulation [15,23].

In contrast to conventional CD4^+^ T cells, CD226 exhibits more intricate functions in regulatory T cells (Tregs). Several studies have shown that CD226 impairs Treg stability and immunosuppressive capacity. Studies in murine models demonstrate that CD226 deficiency in Tregs alleviates insulitis and delays diabetes onset in non-obese diabetic (NOD) mice, suggesting a suppressive function for CD226 in Treg-mediated tolerance during autoimmunity [24]. Similarly, Sato K et al. found that deficiency in CD226 potentiates TIGIT-mediated signaling in Tregs, which maintains Treg cell function and forkhead box P3 (Foxp3) expression in the context of inflammatory settings [25]. However, some studies have come to the opposite conclusion. CD226 is found to play roles in maintaining Treg metabolic fitness and lineage stability via the adenosine monophosphate-activated protein kinase (AMPK)/mammalian target of rapamycin (mTOR)/myelocytomatosis oncogene (Myc) axis [26], and its deficiency impairs Treg immunosuppressive function and induces apoptosis, thereby exacerbating inflammatory responses [27,28]. In T regulatory type 1 cells, CD226 supports proliferation, survival, and IL-10 production, essential for immune tolerance, while CD226 deficiency impairs T regulatory type 1 cell differentiation and STAT5 signaling [29]. These divergent roles of CD226 in different T cell subsets underscore the critical influence of immune cell heterogeneity on therapeutic outcomes, necessitating cell-specific targeting strategies tailored to distinct disease mechanisms and functional pathways.

### 2.2. CD226 and NK Cells

CD226 plays a critical role in the activation and cytotoxic function of NK cells, significantly influencing immune responses against target cells. CD226 enhances NK cell effector functions by facilitating stable interactions with target cells [30]. Its first extracellular domain binds to CD155 and CD112, promoting immune synapse formation and cytotoxicity. Blocking the first extracellular domain disrupts NK cell activation, reducing granule polarization and target cell killing [31]. Mechanistically, CD226 signals through a conserved cytoplasmic motif that recruits Grb2 and activates downstream pathways, including Vav1, PI3K, and phospholipase C gamma 1 (PLC-γ1), leading to actin polymerization and cytotoxic granule release [2]. Additionally, CD226 mediates functional suppression of the transcription factor forkhead box protein O1 (FOXO1), amplifying NK cell cytotoxicity against tumors [32]. Transcriptional profiling and functional studies identifies two NK cell subsets defined by CD226 expression: CD226^+^ NK cells, which exhibit enhanced proliferative activity accompanied by abundant inflammatory cytokine secretion; and CD226^−^ NK cells, which produce higher levels of macrophage inflammatory protein 1. This indicates a CD226-defined axis governing NK cell maturation [33]. In addition, CD226 contributes to the modulation of NK cell education, a process shaped by major histocompatibility complex class I (MHC-I) interactions with inhibitory receptors. Upon target cell recognition, CD226 and LFA-1 rapidly colocalize at the immune synapse, indicating that their coordinated expression is crucial for NK cell education [34]. Notably, CD226 orchestrates NK cell antitumor efficacy by sustaining cytotoxic effector functions and stabilizing immune synapses with malignant cells, making it a pivotal node in cancer immune surveillance. CD226-deficient NK cells exhibit impaired tumor control due to shortened contact durations with cancer cells and reduced cytotoxicity, and antibodies blocking CD226-CD155 interactions suppress NK cell-mediated cytotoxicity [30,35]. Strategies to enhance CD226 signaling can bolster NK cell antitumor activity [32], highlighting CD226 as a promising candidate for cancer immunotherapeutic approaches. While sex hormones are known to modulate various aspects of NK cell function [36], their specific impact on CD226 expression or activity remains unexplored. The potential influence of gender or hormonal status on CD226-mediated NK cell responses represents an intriguing area for future investigation as it could offer valuable insights into observed sex-specific differences in disease susceptibility and therapeutic outcomes involving this pathway.

### 2.3. CD226 and Other Immune Cells

In contrast to its extensively documented functions in T and NK cells, its immunological regulation and effector mechanisms in other immune cell types remain insufficiently explored. CD226 is expressed in macrophage and plays a role in regulating macrophage polarization. CD226 deletion promotes M2 macrophage polarization and inhibits M1 macrophage accumulation, leading to improved post-infarction healing [37]. In obesity, CD226 deficiency in macrophages suppresses phosphorylation of VAV1 and AKT, leading to reduced FOXO1 inactivation. This results in enhanced FOXO1-dependent peroxisome proliferator-activated receptor gamma (PPARγ) expression, which inhibits pro-inflammatory M1 polarization [38]. CD226 expression in macrophages is upregulated during renal fibrogenesis, and its deficiency reduces collagen deposition and pro-inflammatory responses in the kidney. Mechanistically, CD226 promotes M1 macrophage accumulation by suppressing kruppel-like factor 4 (KLF4) expression [39]. In B cells, CD226 is selectively expressed on differentiated subsets, including class-switched memory B cells, plasmablasts, and plasma cells. Upon CpG-ODN (TLR9 agonist) stimulation, CD226 expression is upregulated and functionally contributes to enhanced IL-10 secretion and antibody production in these B cell subsets [40]. A summary of the key effects of CD226 on immune cell function is presented in Table 1.

## 3. The Roles of CD226 in Immune-Mediated Disorders

### 3.1. CD226 and Tumors

#### 3.1.1. CD226 Expression and Prognostic Significance

Elevated CD226 levels in tumor-infiltrating lymphocytes demonstrate a positive association with enhanced anti-tumor activity and favorable prognostic outcomes. In gastric cancer, high CD226^+^CD8^+^ T cell infiltration predicts better survival and response to adjuvant chemotherapy [41,42]. Similarly, CD226 expression on CD8^+^ tumor-infiltrating lymphocytes from colorectal cancer liver metastases serves as an independent prognostic factor, associated with better survival outcomes. IL-15 treatment restores CD226 expression on CD8^+^ TILs, enhancing their functionality and providing a potential therapeutic target [43]. In addition, reduced CD226 expression on peripheral NK cells or tumor-infiltrating lymphocytes is correlated with tumor progression, resistance to immunotherapy, and poor prognosis in gastric cancer, hepatocellular carcinoma, acute myeloid leukemia, pancreatic cancer, melanoma, neuroblastoma, and chronic lymphocytic leukemia [44,45,46,47,48,49,50,51]. Analysis via single-cell sequencing shows that effector CD4^+^ T cells exhibit increased CD226 expression compared to naïve CD4^+^ T cells, while exhausted CD8^+^ T cells display reduced CD226 expression relative to effector CD8^+^ T cells among various types of tumors [52]. Flow cytometry in murine B16F10 melanoma models shows strong correlations between CD226 and the activities of tumor-infiltrating CD8^+^ T cells and NK cells, while gene set enrichment analysis highlights its links to T cell activation, T cell receptor signaling, NK cell-mediated immune responses and cytotoxicity [52]. In addition, CD226 polymorphisms influence cancer risk and treatment outcomes. The rs727088 G allele and rs763361 T allele are correlated with increased susceptibility to gastric cancer and non-small-cell lung cancer [53,54]. In small-cell lung cancer, CD226 rs763361C>T is associated with better chemotherapy response [55].

#### 3.1.2. CD226 and Immune Checkpoint Interactions

The dynamic equilibrium between CD226 and TIGIT modulates effector functions of immune cells, dictating distinct functional outcomes. In tumors where CD226 and TIGIT are not co-expressed, TIGIT dominates via ligand-induced nanoclustering, suppressing cytokine secretion and T cell activation [56]. CD226^high^CD8^+^ T cells exhibit greater responsiveness and self-renewal at tumor sites, and anti-TIGIT treatment enhances their function by promoting CD226 phosphorylation [57]. In melanoma, Tregs exhibit an elevated TIGIT/CD226 ratio, correlating with higher tumor-infiltrating Treg levels and worse clinical prognosis [58]. Similarly, Jin et al. reported an imbalance between CD226 and TIGIT expression on γδ T cells in acute myeloid leukemia patients, with a reduction in CD226^+^ γδ T cells and an increase in TIGIT^+^ γδ T cells, suggesting a potential immune checkpoint barrier contributing to poor prognosis and T cell dysfunction [59].

#### 3.1.3. CD226 in Tumor Immune Evasion

CD226 emerges as a critical immune checkpoint in antitumor immunity, yet tumors subvert its function through CD155-driven degradation mechanisms, enabling evasion of both adaptive and innate immune surveillance. CD155 on tumor cells induces CD226 degradation via phosphorylation and ubiquitination, impairing CD8^+^ T cell function and immunotherapy efficacy [60]. Acute myeloid leukemia blasts reduce CD226 expression on NK cells, suggesting a mechanism of tumor escape by impairing the cytotoxicity of NK cells [47]. Similarly, CD155-expressing hepatocellular carcinoma cells induce CD226 downregulation, impairing CD226-mediated cytotoxicity in both tumor-infiltrating and circulating NK cells, which contributes to the tumor evasion of innate immune surveillance [61].

#### 3.1.4. CD226 as a Therapeutic Target

Emerging preclinical evidence positions CD226 activation as a pivotal amplifier of anti-tumor immunity, synergizing with chemotherapy, immune checkpoint blockade, and vaccine strategies to enhance NK/cytotoxic T lymphocyte (CTL) cytotoxicity across diverse malignancies. In multiple myeloma, CD226 limits spontaneous multiple myeloma development and enhances the efficacy of treatment with cyclophosphamide and bortezomib [62]. CD226 activation or overexpression enhances NK cell cytotoxicity—via granzyme B secretion, apoptosis induction, and efficient degranulation—against diverse malignancies, including triple-negative breast cancer and sarcomas [63,64]. Anti-programmed cell death protein-1 (PD-1) treatment enhances the CD155-CD226 activation pathway, improving NK cell function and inhibiting tumor proliferation [44]. A tumor vaccine expressing both CD226 and Ag85A induces stronger antitumor immunity in a colon carcinoma model, enhancing NK and CTL cytotoxicity and increasing IFN-γ-producing T cells. This suggests that CD226 acts as a genetic adjuvant, synergistically boosting the efficacy of the Ag85A vaccine against colon cancer [65]. Moreover, soluble CD226 exhibit cytotoxic activity against CD155-expressing tumor cells, suggesting its potential as a biotherapeutic agent [66,67]. Exosomes derived from IL-2/IL-15-treated NK cells carry CD226, enabling cytolytic activity at tumor sites and offering a novel immunotherapy strategy [68]. These extensive preclinical evidence underscores CD226’s potential as a promising therapeutic target for cancer immunotherapy.

### 3.2. CD226 and Infectious Diseases

CD226 expression on immune cells is dynamically regulated during infections and correlates with immune responses. In Hantaan virus infection, reduced CD226 expression on inflammatory monocytes is associated with impaired antigen presentation (e.g., CD80 and HLA-DQ/DR/DP downregulation), increased viral load, and disease severity [69]. In latent tuberculosis infection, memory-like NK cells exhibit elevated CD226 expression, which drives their proliferation and effector functions [70]. Similarly, tuberculosis patients show increased CD226^+^ NK and T cell subsets with enhanced production of CD107a and IFN-γ, suggesting CD226 as a biomarker for clinical outcomes and disease progression in tuberculosis [71]. In addition, CD226 polymorphisms influence host susceptibility and disease severity. The rs763362 G/rs727088 G/rs763361 T alleles are linked to severe influenza infections, with higher hospitalization rates in intensive care units. These variants may impair NK cell responses to influenza, exacerbating disease outcomes [72].

Similar to tumor cells, viruses can also exploit CD226-mediated pathways to evade immune surveillance. Human immunodeficiency virus (HIV) persistent infection skews the CD226/TIGIT axis toward TIGIT dominance, suppressing CD8^+^ T cell function. Reduces CD226 expression on CD8^+^ T cells contributes to T cell exhaustion and persistent viral replication, underscoring its role as a checkpoint barrier in HIV cure strategies [73,74]. CD226 blockade significantly compromises the proliferation of Ly49H^+^ NK cells specific for mouse cytomegalovirus and impairs memory NK cell development, while CD226-deficient NK cells shows similar defects [75]. Lymphocytic choriomeningitis virus-infected CD226-deficient mice show delayed viral clearance due to reduced tumor necrosis factor-α (TNF-α) and IL-2 secretion by CD8^+^ T cells, despite intact cytolytic activity [76]. These mechanisms illustrate how pathogens manipulate CD226 to subvert host immunity and how targeting CD226 pathways offers potential strategies to enhance infection control.

### 3.3. CD226 and Allergic Diseases

CD226 plays roles in driving inflammatory responses across diverse immune cells in a variety of inflammatory diseases. CD226 is upregulated in activated CD4^+^ T cells (Th2/Th17 subsets) and type 2 innate lymphoid cells (ILC2s) in asthma patients, enhancing airway hyperreactivity and inflammation. Targeting CD226 reduces ILC2-mediated cytokine secretion (e.g., IL-5, IL-13) and promotes CD4^+^ T cell apoptosis via Caspase-3 activation, ameliorating lung inflammation and airway remodeling in preclinical models [77,78]. In addition, the deficiency of CD226 expression in CD4^+^ T cells alleviates lung inflammation and increases IL-10 levels in ovalbumin-induced allergic asthma [79]. In a murine model of allergic rhinitis, global CD226 knockout mice exhibited reduced nasal symptoms and inflammatory responses, with significant decreases in Th2-inflammatory cytokines, eosinophil recruitment, and goblet cell proliferation [80].

### 3.4. CD226 and Autoimmune Diseases

#### 3.4.1. Rheumatoid Arthritis

CD226 polymorphisms, particularly the rs763361 (Gly307Ser) variant, are significantly associated with rheumatoid arthritis susceptibility across diverse ethnic populations, including Chinese Han, Iranian, and Egyptian cohorts [81,82,83]. Serum soluble CD226 levels correlate with rheumatoid arthritis disease activity, suggesting its utility as a biomarker [81]. Notably, the rs763361 T allele is linked to reduced anti-TNF treatment efficacy, underscoring its potential role in personalized therapeutic strategies [84]. However, CD226 blockade showed limited efficacy in a collagen-induced arthritis murine model [85].

#### 3.4.2. Systemic Lupus Erythematosus

Elevated serum soluble CD226 levels are observed in active systemic lupus erythematosus and correlate with disease activity indices and anti-dsDNA antibody titers, positioning soluble CD226 as a biomarker to monitor disease flares [86]. CD226^+^ B cells, particularly switched-memory subsets, are increased in systemic lupus erythematosus patients and correlated with higher disease activity [87]. Additionally, the CD226 rs763361 polymorphism is linked to elevated risk of systemic lupus erythematosus, particularly in Chinese Han populations [88]. However, CD226^+^ NK cells are deficient in active systemic lupus erythematosus, potentially due to activation-induced cell death [89].

#### 3.4.3. Systemic Sclerosis

In systemic sclerosis patients’ skin tissue, CD226 overexpression exacerbates fibrotic progression by enhancing T cell infiltration and prompting the secretion of pro-inflammatory cytokines including TNF-α and IL-6 [90]. In systemic sclerosis patients, elevated CD226 expression in CD8^+^ T cells promotes profibrotic IL-13 production and endothelial damage [91]. Murine studies demonstrate that CD226 neutralization reduces dermal fibrosis by suppressing cytokine production and T cell activation [90]. Genome-wide association analyses in European ancestry cohorts have identified the CD226 rs763361 T allele as a genetic risk locus for systemic sclerosis, demonstrating particularly strong associations with severe clinical phenotypes including diffuse cutaneous involvement [92]. However, CD226 polymorphisms demonstrate no significant association with systemic sclerosis susceptibility in Iranian populations, highlighting ethnic variability in genetic risk [93].

#### 3.4.4. Primary Sjögren’s Syndrome

In primary Sjögren’s syndrome, CD226 expression is significantly altered in immune cells. Elevated frequencies of CD4^+^ T cells with CD226 and TIGIT expression show significant association with clinical disease severity, suggesting their potential as therapeutic targets [94]. Additionally, increased proportions of CD226^+^CD14^+^ monocytes have been reported in primary Sjögren’s syndrome patients, correlating with the severity of the disease [95]. Intriguingly, plasma soluble CD226 levels are reduced in primary Sjögren’s syndrome and inversely correlate with clinical manifestations and disease progression, indicating its diagnostic potential [96].

#### 3.4.5. Type 1 Diabetes

Genetic research has identified the CD226 rs763361 polymorphism as a risk factor for type 1 diabetes, particularly in Chinese Han children and Brazilian populations [97,98]. Functional studies in NOD mice models have shown that CD226 deficiency or blockade delays insulitis onset and reduces diabetes incidence by inhibiting effector T cell activation and enhancing Treg function [99,100]. CD226^+^ CD8^+^ T cell subsets exhibit a enhanced cytotoxic activity and are inversely correlated with β-cell function. CD226 blockade delays the onset of insulitis and mitigates the severity of hyperglycemia in both streptozotocin-induced and cyclophosphamide-induced murine diabetes models [101].

#### 3.4.6. Multiple Sclerosis and Neuromyelitis Optica

An increase in soluble CD226 levels has been detected in multiple sclerosis and neuromyelitis optica patients, correlating with neuroinflammation and disease progression [102]. CD226 deficiency in experimental autoimmune encephalomyelitis models reduces Th17 infiltration and increases Treg suppressive capacity, attenuating disease severity [103,104,105]. In neuromyelitis optica, CD226 overexpression on T regulatory type 1 cells correlates with disease severity, suggesting its role as a progression biomarker [106]. The CD226 Gly307Ser (rs763361) polymorphism is correlated with a higher risk of neuromyelitis optica in Southern Han Chinese, but not with multiple sclerosis [107].

#### 3.4.7. Other Autoimmune Diseases

Antiphospholipid syndrome (APS) is a complex autoimmune disorder characterized by the presence of antiphospholipid antibodies [108]. These antibodies initiate signaling through receptors like toll-like receptor 4 (TLR4), low-density lipoprotein receptor-related protein (LRP) 6, and LRP8, and this process relies on lipid rafts—cholesterol- and sphingolipid-rich microdomains within the plasma membrane [108,109]. Notably, CD226 has been reported to localize within lipid rafts [110], and this spatial co-localization strongly implies a potential mechanistic link between APS and CD226 function. Exploring whether CD226 function and localization depend on lipid raft integrity could open new avenues for understanding APS pathogenesis. Methyl-β-cyclodextrin, with its ability to selectively disassemble lipid rafts [109], is a valuable experimental tool. In addition, the imbalance in the expression of CD226/TIGIT in NK cells is associated with antiphospholipid syndrome progression and autoantibody status, suggesting a potential therapeutic target [111].

Furthermore, CD226 has also been found to play roles in other autoimmune diseases. The CD226 rs763361 polymorphism demonstrates a significant correlation with increased risk of juvenile idiopathic arthritis across various subtypes [112]. Enhanced activation of the CD155-CD226 signaling pathway is observed in inflamed muscle tissues of patients with idiopathic inflammatory myopathies, correlating with disease severity and muscle damage [113]. Furthermore, the CD226 rs763361 polymorphism is linked to higher susceptibility to autoimmune thyroid disease and psoriasis [114,115]. These findings highlight the broad role of CD226 in autoimmune pathogenesis. The multifaceted roles of CD226 in the pathogenesis and progression of various immune-mediated disorders are summarized in Table 2.

## 4. Perspectives

The extensive role of CD226 in immune regulation underscores its therapeutic potential and complexity. The dual functionality of CD226 in Treg functionality highlights its complex contribution to immune dysregulation and underscores the need for cell-specific targeting strategies. Future research should delve deeper into the molecular mechanisms underlying CD226’s diverse functions in different immune cell types and such understanding will contribute to the development of more targeted therapeutic interventions. CD226 polymorphisms significantly impact disease susceptibility and progression, highlighting the potential of personalized medicine approaches targeting CD226. Additionally, developing precise therapeutic interventions that target CD226’s context-dependent functions without causing systemic immune dysregulation remains a critical challenge. Combining CD226-targeted therapies with existing immunotherapies may also offer synergistic benefits, warranting further exploration in clinical settings.

## Figures and Tables

**Table 1:** Effects of CD226 in immune cells.

Cell Type	Roles of CD226
Conventional T cells	Enhances CD8^+^ T cell maturation and memory formation [16,17]; promotes Th1/Th17 polarization via enhanced IL-17/IFN-γ production [18,19]; facilitates early Tfh differentiation and proliferation [21]
Treg cells	Dual roles: impairs stability/suppressive function [24,25] vs. maintains metabolic fitness/lineage stability [26]
NK cells	Enhances activation, cytotoxicity and stable target cell interactions [30,31]; modulates NK cell education [34]
Macrophages	Drives M1 polarization [37,38,39]

**Table 2:** The roles of CD226 in immune-mediated disorders.

Disease Category	Disease/Context	Roles of CD226
Tumor	Multiple Tumor Types	High CD226^+^ TILs infiltration correlate with better prognosis [41,42,43,44,45,46,47,48,49,50,51]; tumor-induced CD226 downregulation promotes immune evasion [60,61]; polymorphisms increase cancer susceptibility and influence treatment response [53,54,55]
Infectious Diseases	Viral Infections	Reduced expression on immune cells impairs antiviral immunity [69,73,74,75,76]
	Tuberculosis	Elevated expression on NK/T cells enhances effector functions [70,71]
Allergic Diseases	Asthma	Upregulation in Th2/Th17/ILC2s drives inflammation [77,78]
	Allergic Rhinitis	Deficiency reduces Th2 cytokines, eosinophil recruitment, and inflammatory responses [80]
Autoimmune Diseases	Rheumatoid Arthritis	rs763361 polymorphism confers susceptibility [81,82,83]; soluble CD226 correlates with disease activity [81]
	Systemic Lupus Erythematosus	Increased soluble CD226 and CD226^+^ switched-memory B cells correlate with disease activity [86,87]; rs763361 polymorphism increases susceptibility [88]
	Systemic Sclerosis	Overexpression in skin tissue/CD8^+^ T cells drives pro-inflammatory cytokines secretion [90,91]; rs763361 T allele is genetic risk in Europeans [92]
	Primary Sjögren’s Syndrome	Increased proportions of CD226^+^ CD4^+^ T cells/monocytes correlate with disease severity [94,95]; reduced soluble CD226 inversely associates with disease progression [96]
	Type 1 Diabetes	rs763361 polymorphism increases susceptibility [97,98]; blockade delays insulitis onset [99,100,101]
	Multiple Sclerosis and Neuromyelitis Optica	Increased soluble CD226 correlates with neuroinflammation [102]; CD226 deficiency attenuates experimental autoimmune encephalomyelitis severity [103,104,105]
